# Parental behaviors regarding fever in young children in Benin: study of population survey data

**DOI:** 10.11604/pamj.2024.47.125.41320

**Published:** 2024-03-21

**Authors:** Gbènonminvo Enoch Cakpo, Gountante Kombate, Matè Alonyenyo Labité, Komi Ameko Azianu, Mazimna M'belou

**Affiliations:** 1 Institut Supérieur des Sciences de la Population, Université Joseph Ki-Zerbo, Ouagadougou, Burkina Faso,; 2 Centre de Recherche et d´Appui en Statistique, Parakou, Bénin,; 3 Society for Study and Research in Public Health, Ouagadougou, Burkina Faso,; 4 Ecole Nationale des Auxilliaires Médicaux de Lomé, Lomé, Togo

**Keywords:** Fever, care-seeking, children under five, Benin

## Abstract

**Introduction:**

the search for care of parents in case of the febrile episode of children is not always systematic. This study aims to improve knowledge on health care research in cases of fever in children under five years of age in Benin.

**Methods:**

this study used data from the Benin Demographic and Health Survey 2017-2018. Counselling or seeking care is defined as any child under 5 years of age who has a fever in the two weeks prior to the interview. Univariate and multivariate logistic regression analyses were performed using generalized linear model.

**Results:**

a total of 2465 children were surveyed. The model predicting seeking appropriate advice or care in febrile children in Benin was distance from the nearest health center, region, maternal age, and socioeconomic status. Indeed, febrile children whose mothers perceived difficult geographical access to the health center were 30% less likely to seek care, compared to children whose geographical access to the health center was easy (aOR=0.70 (0.54-0.90)). In addition, mothers living in the Hill region were more likely (AOR=5.73 (3.53-9.45)) to seek appropriate advice or care compared to those living in Alibori. In terms of socioeconomic status, children whose mothers were very wealthy were more likely to have their mothers seek care (aOR=1.93 (1.33-2.81))

**Conclusion:**

interventions to improve universal primary health care coverage in terms of geographic accessibility, awareness and health literacy are the best allies for routine care.

## Introduction

Fever is not a disease, but rather a sign of an illness. Most of the time, fever indicates that the body is battling a viral or bacterial infection. It is manifested by an increase in the internal temperature in order to fight the virus or bacteria. This makes it the first reason for consultation and hospitalization of children in Africa [1]. It represents the main symptom of frequent pathologies in children and is classified as one of the leading causes of morbidity in children in developing countries [2]. Globally, infectious diseases, including pneumonia, diarrhea and malaria, characterized by fever as a primary sign, remain the leading causes of death among children under 5 years of age [3]. Seeking care is the best way to treat and prevent complications. Parents' search for medical care in the event of a febrile episode of children is not always systematic. The management of fevers at home requires early diagnosis, and often inadequate treatment [1]. The cost of treatment, socio-economic status; the level of education, the proximity of health facilities, the accessibility to trained providers; the availability of transport and the level of knowledge could be at the origin of this late use of care [4,5]. In developing countries, particularly in sub-Saharan Africa, according to household surveys, the search for fever care is still at a more or less satisfactory level. It is 53.9% in Benin in 2017; 57.8% in Mali in 2018; 9.5% in Ghana in 2019; 73.8% in Burkina Faso in 2017 [6]. In Benin, malaria accounts for 44.9% of the causes of use of care in health facilities and is the leading condition whose first signs are characterized by fever [7]. The national incidence is 185 cases per 1000 inhabitants in 2018 especially among pregnant women and children under five years of age [8]. In light of the public health problem posed by the search for care in the event of fever, the study of parental recourse to care in the event of a febrile episode in children is of paramount importance, since the control of its determinants could strengthen interventions related to prevention and health promotion. Thus, this study aims to describe the care-seeking behaviors of mothers of febrile children under the age of five in Benin, and to determine the factors associated with children, households and the community.

## Methods

**Figure 1:**
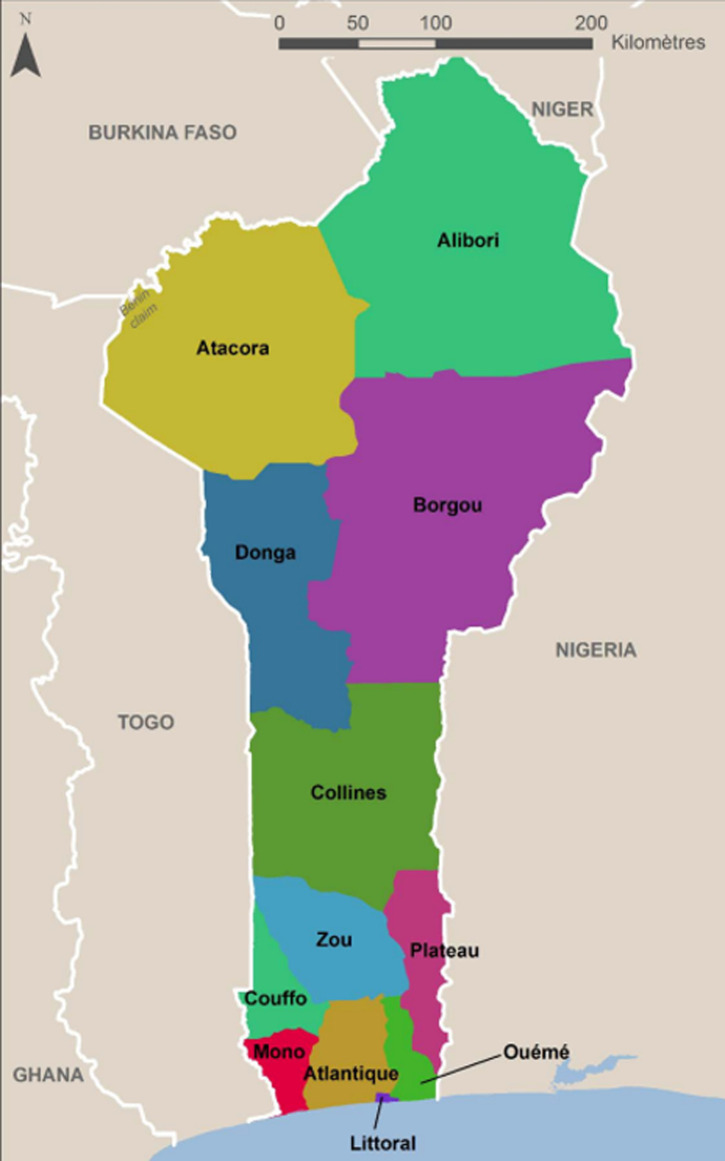
administrative map of Benin

**Scope of the study:** Benin, a West African country, has a total population that has increased from 10,008,749 in 2013 [9] to 12,451,031 in 2021 [10]. Covering an area of 114,763 square kilometers, Benin is bounded to the south by the Atlantic Ocean, to the west by Togo, to the north by Burkina Faso and Niger and to the east by Nigeria. At the administrative level, Benin has had 12 departments (Figure 1) since 15 January 1999, in accordance with Law No. 97-028 on the organization of the territorial administration of the Republic of Benin [7]. Benin has a hot and humid climate, conducive to the spread of disease vectors and is partly responsible for the national epidemiological profile dominated by infectious and parasitic diseases. Benin's health system is organized according to a three-tier pyramidal structure. The central level constituted by the Office of the Minister of Health and the Central and Technical Directorates. The intermediate level is represented by the Departmental Directorate of Health (DDS) which is responsible for the implementation of the health policy defined by the Government, the programming and coordination of all the activities of the health services at the departmental level. The peripheral level is represented by the Health Zone Management Team which is in charge of coordinating and monitoring the implementation of the operational work plan at the level of the health zone [7,11]. In 2018, Benin had 1371 health facilities, of which 437 were private and 934 were public [9].

**Data source and sampling:** the present study used data from the Demographic and Health Survey in Benin (EDSB-V) 2017-2018. This survey used a nationally representative sample, covering all 12 departments in both urban and rural areas. The sample consisted of 14,435 households (6,528 urban in 251 clusters and 7,907 rural households in 304 clusters) were selected. Of these households, 14,156 (6,364 in urban areas and 7,792 in rural areas) were actually surveyed. During this study, all women aged 15-49 who usually lived in the selected households and were present the night before the survey were eligible to be interviewed. A total of 15,928 women aged 15-49, including 7,045 in urban areas and 8,883 in rural areas, were included in the study [12].

**Data collection:** data collection work was conducted from November 6, 2017, to February 28, 2018. The women's individual questionnaire consists of 15 sections that estimate socio-economic and demographic indicators including: educational attainment, fertility indicators, fertility determinants, family planning, child mortality indicators, maternal health, child health, breastfeeding and nutrition, malaria, HIV/AIDS and STIs, other health problems, the status of women, as well as household and sexual activity. It should be noted that the care-seeking behavior for children under 5 years of age with fever in the 2 weeks prior to the interview was requested.

**Study variables:** the study-dependent variable was the search for care or advice in febrile children under 5 years of age. This variable takes modality 1 (Yes) if advice or care has been sought from a health professional in public or private health centers, including community health workers. Otherwise, it takes modality 0 (No) if advice or care has been sought from itinerant vendors, pharmacies, shops, markets, families, friends, traditional healers or no advice at all. The independent variables were the age, sex of the child, whether the child was breastfeeding at the time of the survey, the mother's age, her level of education, the place of residence, the region, the distance to the nearest health center, marital status, the household wealth index, occupation, religion, ethnic origin and gender of the head of household. The wealth index was constructed using principal component analysis using information on household assets, including the ownership of a number of consumer goods and housing characteristics.

**Data analysis:** in order to achieve the research objectives, several statistical methods were used. Initially, the analysis consisted mainly of a description of the differential prevalence of fever in children under five years of age. Then, cross-tabulations between the dependent variable and the independent variables were produced, and their statistical associations were analyzed. Univariate and multivariate binary logistic regression were performed to identify factors associated with appropriate healthcare or advice seeking behaviors for childhood fever. Crude odds ratios (ORs) and adjusted odds ratios (aOR) were estimated to assess the strength of associations and used 95% confidence intervals for significance tests. The data were analyzed using version 4.0.4 of the R software.

**Ethics approval and consent to participate:** the Demographic and Health Survey of Benin (EDSB) 2017-2018 was implemented by the National Institute of Statistics and Economic Analysis (INSAE). It had ethical approval and informed consent from the parent and/or legal guardian. Ethical approval was obtained by INSAE from the National Health Research Ethics Committee and the International Coach Federation (ICF International) Ethics Committee before collection began. Therefore, informed consent from the parent and/or legal guardian or additional ethical approval was not required for this secondary data analysis. However, the DHS program did give permission to download and use the data from DHS. As a result, individual-level and aggregate community variables did not include personal identifiers such as names, house numbers, and telephone numbers. However, it should be noted that ethical considerations regarding the use of human subjects were strictly adhered with, and all methods were performed in accordance with the Declaration of Helsinki.

## Results

**Figure 2:**
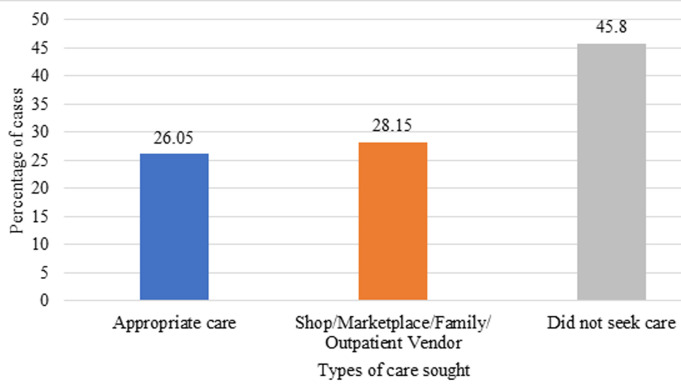
distribution of the study population by the different types of care sought

**Characteristics of the study population:** the study population consisted of children under five years of age who had a fever in the two weeks preceding the survey. In total, the survey involved 2,465 children. Boys (51.97%) were more represented than girls (48.03%), most children were between 12 and 23 months of age (Table 1). The largest group of children in the sample (21.78%) came from the Poor Wealth Index, while only 15.74% came from the Richest Wealth Index. The majority (65.35%) lived in rural areas (Table 1.1). Among these children, appropriate care was requested in 28.15% of cases (Figure 2).

**Table 1:** study population

Variable		
**Age**	**Actual**	**%**
0-11	496	20.12
12-23	592	24.02
24-35	573	23,25
36-47	403	16.35
48-59	401	16.27
**Sex of children**		
Male	1281	51.97
Female	1184	48.03
**Household head's sex**		
Male	2055	83.37
Female	410	16.63
**Current breastfeeding**		
Not	984	39.92
Yes	1481	60.08
**Place of residence**		
Urban	854	34.65
Rural	1611	65.35
**Distance to the nearest health**		
Not big problem	1206	48.92
Big problem	1259	51.08
**Region**		
Alibori	390	15.82
Atacora	185	7,51
Atlantic	396	16.06
Borgou	213	8.64
Collines	135	5.48
Couffo	141	5.72
Donga	135	5.48
Littoral	106	4,3
Mono	118	4.79
Ouémé	213	8.64
Plateau	235	9.53
Zou	198	8.03

**Table 1.1:** study population

Variable	Actual	%
**Age of respondent**		
15-24	658	26.69
25-34	1309	53.1
35-49	498	20.2
**Level of education**		
No schooling	1642	66.61
Primary	609	24.71
Secondary+	214	8.68
**Status socio eco**		
Poorest	487	19.76
Poorer	537	21.78
Middle	528	21.42
Richer	525	21.3
Richest	388	15.74
**Matrimonial status**		
Single	59	2.39
Married	2319	94.08
Divorced	87	3.53
**Occupation**		
Work	1566	63.53
Not working	899	36.47
**Religion**		
Animist	442	17.93
Christian	1288	52.25
Muslim	735	29.82
**Ethnic**		
Adja and related	316	12.82
Bariba and related	285	11.56
Dendi and related	118	4.79
Fon and related	922	37.4
Yoa, lokpa and related	64	2.6
Betamaribe and related	180	7.3
Peulh and related	219	8.88
Yoruba and related	273	11.08
Thether	87	3.53

**Search for care or advice and associated factors:** in Table 2 and Table 2.1 presents the results of bivariate and multivariate analyses of the search for care for children's fever in the two weeks preceding the survey. These analyses show that in 27.75% of febrile children, appropriate care was sought in the group of children living in urban areas. On the other hand, in rural areas, in 71.63% of cases, care in the event of a febrile episode was not sought. The results show that children from a very wealthy family had a high propensity (34.02%) to receive care, followed by children from wealthy families (32.38%). Children whose mothers had a secondary and higher education level had a high propensity (39.25%) to seek appropriate care, followed by those with no education (27.34%). For the region, 80.19% of children whose mothers resided in the Littoral did not seek medical attention for fever. After bivariate analyses, distance from the nearest health center, mother's education, wealth index, region, ethnicity, maternal age, breastfeeding and marital status were strongly associated and significant with the use of appropriate counselling or care. No association was observed with the child's age, sex, place of residence, occupation, religion and sex of the head of household. The final model predicting seeking appropriate advice or care in febrile children in Benin was distance from the nearest health center, region, age of mother, and socioeconomic status. Febrile children whose mothers perceived difficult geographical access to the health centre were 30% less likely to seek care, compared to children whose geographical access to the health center was easy (aOR=0.70 (0.54-0.90)). It should also be noted that a regional difference was observed in the search for appropriate advice or care. Mothers living in the Collines region were more likely (aOR=5.73 (3.53-9.45) 0.0001)) to seek appropriate advice or care compared to those living in Alibori. Women aged 35 to 49 were more likely (aOR=1.37 (1.03 -1.82) 0.030) to seek appropriate advice or care compared to younger women (15 to 24 years). In terms of socioeconomic status, children whose mothers were very wealthy were more likely to have their mothers seeking care (aOR=1.93 (1.33-2.81)) compared to those whose wives were very poor. It should also be noted that those with poor mothers were also more likely (aOR=1.49 (1.11-2.01)) to seek appropriate advice or care compared to children with very poor mothers

**Table 2:** factors associated with seeking appropriate care or advice

N=2465	Appropriate care	%	Inappropriate care	%	Crude OR (95% CI) P.value	Adjusted OR (95% CI) P Value
**Age**						
0-11	129	26.01	367	73.99	Ref.	-
12-23	137	23.14	455	76.86	1.14 (0.88-1.47) ns	-
24-35	126	21.99	447	78.01	0.94 (0.72-1.24) ns	-
36-47	128	31.76	275	68.24	0.93 (0.70-1.22) ns	-
48-59	174	43.39	227	56.61	1.02( 0.76-1.39) ns	-
**Sex of children**						
Male	378	29.51	903	70.49	Ref.	
Female	316	26.69	868	73.31	1.13 (0.95-1.35) ns	-
**Household head’s sex**						
Male	569	27.69	1486	72.31	Ref.	-
Female	125	30.49	285	69.51	0.96 (0.76-1.22) ns	
**Current breastfeeding**						
Not	293	29.78	691	70.22	Ref.	Ref.
Yes	401	27.08	1080	72.92	1.19 (0.99-1.42) *	0.92 (0.76-1.12) ns
**Place of residence**						
Urban	237	27.75	617	72.25	Ref.	
Rural	457	28.37	1154	71.63	0.95 (0.79-1.15) ns	-
**Region**						
Alibori	39	10.00	351	90.00	Ref.	Ref.
Atacora	55	29.73	130	70.27	0.26 (0.16-0.41) ***	3.32 (2.07 – 5.37) ***
Atlantic	116	29.29	280	70.71	0.25 (0.16-0.38) ***	2.66 (1.70-4.22) ***
Borgou	63	29.58	150	70.42	0.30 (0.18-0.47) ***	2.88 (1.81-4.64) ***
Collines	67	49.63	68	50.37	0.12 (0.07-0.19) ***	5.73 (3.53-9.45) ***
Couffo	46	32.62	95	67.38	0.27 (0.16-0.44) ***	2.77 (1.65-4.69) ***
Donga	64	47.41	71	52.59	0.11 (0.07-0.18) ***	6.67 (4.08 -11.06) ***
Littoral	21	19.81	85	80.19	0.46 (0.27-0.78) ***	1.10 (0.60-2.01) ns
Mono	32	27.12	86	72.88	0.32 (0.19-0.54) ***	2.42 (1.41-4.17) ***
Ouémé	51	23.94	162	76.06	0.37 (0.23-0.60) ***	1.90 (1.14-3.17) **
Plateau	66	28.09	169	71.91	0.31 (0.20-0.49) ***	2.50 (1.58-4.01) ***
Zou	74	37.37	124	62.63	0.20 (0.12-0.31) ***	3.84 (2.40-6.21) ***

**Table 2.1:** factors associated with seeking appropriate care or advice

N=2465	Appropriate care	%	Inappropriate care	%	Crude OR (95% CI) P value	Adjusted OR (95% CI) P value
**Age of respondent**						
15-24	162	24.62	496	75.38	Ref.	**Ref**.
25-34	380	29.03	929	70.97	0.80 (0.64-1.01) **	1.31 (1.04-1.66) **
35-49	152	30.52	346	69.48	1.19 (0.99-1.42) *	1.37 (1.03 -1.82) **
**Level of education**						
No schooling	449	27.34	1193	72.66	Ref.	Ref.
Primary	161	26.44	448	73.56	0.90 (0.71-1.13) ns	0.93 (0.72-1.19) ns
Secondary+	84	39.25	130	60.75	0.63 (0.49-0.81) ***	1.31 (0.98 -1.75) ns
**Status socio eco**						
Poorest	105	21.56	382	78.44	Ref.	Ref.
Poorer	161	29.98	376	70.02	0.63 (0.47- 0.84) ***	1.49 (1.11-2.01) ***
Middle	126	23.86	402	76.14	0.78 (0.58-1.04) *	1.07 (0.78-1.46) ns
Richer	170	32.38	355	67.62	0.53 (0.39-0.70) ***	1.59 (1.17-2.18) ***
Richest	132	34.02	256	65.98	0.51 (0.38-0.69) ***	1.93 (1.33-2.81) ***
**Matrimonial status**						
Single	20	33.90	39	66.10	Ref.	Ref.
Married	657	28.33	1662	71.67	1.33 (0.74-2.31) ns	1.21 (0.44-1.20) ns
Divorced	17	19.54	70	80.46	2.21 (1.01-4.86) *	1.4 (0.80-2.22) ns
**Occupation**						
Work	437	27.91	1129	72.09	Ref.	-
Don't work	257	28.59	642	71.41	0.88 (0.69-1.13) 0.80 ns	-
**Religion**						
Animist	129	29.19	313	70.81	Ref.	-
Christian	398	30.90	890	69.10	0.84 (0.66-1.07) 0.22 ns	-
Muslim	167	22.72	568	77.28	1.14 (0.87-1.49) 0.10 ns	-
**Ethnic**						
Adja and related	92	29.11	224	70.89	Ref.	
Bariba and related	48	16.84	237	83.16	1.80 (1.21-2.69) ***	0.88 (0.43-1.77) ns
Dendi and related	27	22.88	91	77.12	1.10 (0.68-1.81) ns	0.68 (0.31-1.52) ns
Fon and related	279	30.26	643	69.74	0.89 (0.66-1.18) ns	0.94 (0.59-1.49) ns
Yoa, lokpa and related	19	29.69	45	70.31	0.86 (0.47 -1.61) ns	1.90 (0.79-4.67) ns
Bbetamaribe and related	66	36.67	114	63.33	0.68 (0.46-1.02) ns	0.57 (0.26-1.23) ns
Peulh and related	38	17.35	181	82.65	1.72 (1.14-2.65) ***	1.16 (0.57-2.33) ns
Yoruba and related	93	34.07	180	65.93	0.75 (0.53-1.06) ns 0.10684	0.84 (0.46-1.50) ns
Other	32	36.78	55	63.22	0.56 (0.35-0.90) ***	0.93 (0.44-1.98) ns

## Discussion

This study was carried out with the aim of evaluating care-seeking behaviors among febrile children in Benin and identifying the associated factors. To do this, data from the 2017-18 Demographic and Health Survey were used. These are data collected from households, representative at national level and therefore the results could be generalized at national level. The results show that seeking care among febrile children in Benin was strongly associated with the region, the age of the respondent, the mother's level of education and the distance from the child's household and the nearest health center. The results clearly demonstrate significant differences in the search for fever care by geographic region. Indeed, mothers of children living in the regions of Atacora, Atlantic, Borgou, Collines, Couffo de Donga de Mono de Oumo du Plateau and Zou are more likely to seek care from health professionals than those living in the Alibori region. These geographical differences were observed in the studies of M. Negatou and al (2021) in Burkina Faso [13] and Kombate and al in Togo [14]. This regional difference, observed, is probably related to cultural differences. Indeed, according to A. Franckel and al (2008), the geographic location of populations is associated with their exposure to different cultural influences and which are associated with significantly different care practices [15,16]. Based on household economic conditions, the socio-economic status of the household was significantly associated with seeking care for fever in children. The search for appropriate health care in case of fever in children increases with the evolution of the household wealth index. These results are similar to those found in Burkina Faso [13] and Uganda [17].

These studies found that the search for appropriate care for fever became weaker and weaker as one moved from the least poor to the poorest [17]. Even in the presence of free interventions directed towards the most disadvantaged households in Benin, it is noted that the poorest still have a comparative disadvantage in access to care. This embedding could be related to other factors, including distance from the nearest health facility, road quality, access to transport and waiting times [13]. As a result, the respondent's age was significantly associated with seeking care. Women aged 35 to 49 were more likely (aOR=1.37 (1.03 -1.82)) to seek appropriate advice or care compared to younger women (15 to 24 years). This could be explained by the fact that older women would have more experience in what to do in the event of an episode of fever in children, following information sessions and experiences during first-time. Households' perception of the distance between them and the nearest health center shows that children belonging to households who consider this distance to be a big problem are 30% less likely to seek care for fevers, compared to those whose distance is not a problem. Even if efforts were made, the characteristics of the health facilities themselves, including the geographical proximity of households, were recognized in Malawi as important deterrents that prevent many people from seeking health care for children under five [18]. To enable children to receive not only timely but also adequate treatment, the private sector may need to be included in improving the quality of care [19]. On the other hand, in Benin, the involvement of the private sector “is not yet sufficient to effectively stem the phenomenon of healers and street vendors of medicines who are sometimes the only providers accessible to the poorest populations and especially those living in rural areas” [20]. This highlights the importance of closely examining the impact of the geographical and financial accessibility of care on household behavior in order to improve the quality of care of children. Against all odds, the results show the mother's level of education does not explain the seeking care behavior. In Mozambique, Cassy A and al (2019) have shown that there is a relationship [21].

**Limitations:** it should be noted that this study had some important limitations. Indeed, the study did not explore certain personal and behavioral factors such as mothers' knowledge, attitude, beliefs, and perceptions regarding fever and seeking health care that are known to influence behaviors. In addition, the data used for fever and care use of caregivers were self-reported. As a result, some respondents may have difficulty remembering all the relevant details. They can also be influenced by a social desirability bias. However, this recall bias was minimized in the present study, as only fever episodes observed in children in the last two weeks prior to the surveys were considered.

## Conclusion

This study identified determinants related to care-seeking in the event of a febrile episode in children under five years of age two weeks before the survey in Benin. The results confirmed that the search for appropriate care for fever is not systematic for children. Appropriate care is sought in 28.15% of cases. The final predictor of seeking appropriate advice or care was distance from the nearest health center, region, age of mother, and socioeconomic status. Interventions to improve universal primary health care coverage in terms of geographic accessibility, awareness-raising and health education are the best allies for the use of routine care. The regional differences highlighted are additional considerations for such interventions. Studies of certain personal and behavioral factors such as mothers' knowledge, attitudes, beliefs and perceptions of fever and seeking health care will provide a better understanding of appropriate advice or behavior in children to seek care for fever.

### What is known about this topic

- *Parental behaviour in the case of fever in young children can have implications for public health, particularly in terms of under-use of healthcare services, which can delay appropriate treatment of the illness;*
- *There are cultural beliefs that can influence parents' decisions about managing fever in their children, such as a preference for traditional remedies over seeking appropriate care*.

### What this study adds

- *The study highlights the low use of appropriate care by mothers of children; Indeed, only 28.15% of mothers sought appropriate care for their febrile child; this suggests that seeking appropriate care for fever is not systematic among children;*
- *The regional differences identified in this study are important factors to consider when planning interventions to improve mothers' search for appropriate care;*
- *The perception of the distance between households and the nearest healthcare facility is crucial to access to care for children with fever; children from households where distance is perceived as a significant problem are less likely to receive appropriate care than those for whom distance is not a barrier; although efforts are being made to improve access to care, the characteristics of the health facilities themselves, including the geographical proximity of households, are recognised as significant deterrents to many people seeking health care*.
